# Turn on Fluorescence Sensing of Zn^2+^ Based on Fused Isoindole-Imidazole Scaffold

**DOI:** 10.3390/molecules27092859

**Published:** 2022-04-30

**Authors:** Sutapa Sahu, Yeasin Sikdar, Riya Bag, Javier Cerezo, José P. Cerón-Carrasco, Sanchita Goswami

**Affiliations:** 1 Department of Chemistry, University of Calcutta, 92, A.P.C. Road, Kolkata 700009, India; sutapa.sahu11@gmail.com (S.S.); riyabag.chem@gmail.com (R.B.); 2 Department of Chemistry, The Bhawanipur Education Society College, 5, LalaLajpat Rai Sarani, Kolkata 700020, India; y.sikdar@gmail.com; 3 Departamento de Química, Universidad Autónoma de Madrid, 28049 Madrid, Spain; javier.cerezo@uam.es; 4 Centro Universitario de la Defensa, Academia General del Aire, Universidad Politécnica de Cartagena, C/Coronel López Peña S/N, Santiago de La Ribera, 30720 Murcia, Spain

**Keywords:** isoindole, imidazole, chemosensors, zinc ion, fluorescence

## Abstract

Optical chemosensors caused a revolution in the field of sensing due to their high specificity, sensitivity, and fast detection features. Imidazole derivatives have offered promising features in the literature as they bear suitable donor/acceptor groups for the selective analytes in the skeleton. In this work, an isoindole-imidazole containing a Schiff base chemosensor (1-{3-[(2-Diethylamino-ethylimino)-methyl]-2-hydroxy-5-methyl-phenyl}-2H-imidazo[5,1-a]isoindole-3,5-dione) was designed and synthesized. The complete sensing phenomena have been investigated by means of UV-Vis, fluorescence, lifetime measurement, FT-IR, NMR and ESI-MS spectroscopic techniques. The optical properties of the synthesized ligand were investigated in 3:7 HEPES buffer:DMSO medium and found to be highly selective and sensitive toward Zn^2+^ ion through a fluorescence turn-on response with detection limit of 0.073 μm. Furthermore, this response is effective in gel form also. The competition studies reveal that the response of the probe for Zn^2+^ ion is unaffected by other relevant metal ions. The stoichiometric binding study was performed utilizing Job’s method which indicated a 1:1 sensor–Zn^2+^ ensemble. Computational calculations were performed to pinpoint the mechanism of sensing.

## 1. Introduction

Metal ions are deeply rooted in both environment and biological systems [1,2,3,4]. Although required in many fundamental processes, their quantitative and qualitative analysis is a critical measure because negative effects are associated if present in excess. In that framework, optical chemosensors are the easiest way to detect analytes as they open the door for cost–effective and fast detection methods [5,6,7]. Among optical chemosensors, fluorescent species are widely used for assessing ions due to their selectivity, high sensitivity, and low detection ability [8,9,10].

The present contribution brings the focus towards the detection of Zn^2+^. That ion is mostly found in biological systems along with other metal ions including Fe^3+^, Mg^2+^, Mn^2+^ [11,12]. However, Zn^2+^ specifically participates in a wide range of cellular functions in human body, e.g., cofactor of carbonic anhydrase and zinc-finger proteins (ZNFs), DNA synthesis, RNA transcription, regulation of metalloenzymes, neurophysiology, and apoptosis [13,14,15,16,17]. Zn^2^^+^ also plays a core role in brain functionality, a region that accumulates the highest concentration (150 mM–300 nM) as part of the synaptic transmission machinery [18,19,20,21,22,23,24,25]. Other fundamental biological actions of Zn^2+^ have been detected in immune system and cell growth, cell division, proper development of neutrophils, and as natural killer cells [26,27,28,29,30,31]. As expected, negative effects appear if Zn^2+^ is out of the natural ratio. Low levels of Zn^2+^ might lead to serious health concerns such us difficulty in wound healing, alopecia, diarrhea, poor growth, dysfunction of the immune and nervous system with congenital abnormalities and blood disorders, to cite only a few [32]. On the contrary, accumulation of Zn^2+^ in our bodies might cause anemia, epilepsy, and Alzheimer’s disease [33]. As other ions, Zn^2+^ also impacts into environment, so that its bioavailability in soil must be monitored. For instance, it has been shown that an excess in Zn^2+^ concentration induces phytotoxic effect that are concomitant with the degradation of crop quality, which in turn indirectly affects the human life [34,35,36,37]. Unfortunately, due to the filled d orbital of Zn^2+^, its visual detection is still scarce [38,39]. The available chemosensors for Zn^2+^ are limited to naphthalene [40], quinoline [41,42], coumarin [43], rhodamine [44], flavonol [45], benzothiazole [46], phenanthroline [47], julolidine [48] building blocks. The determination of Zn^2+^ remains an essential issue to ensure that its concentration is tuned at the optimal biological and environmental level.

Up until now, the use of imidazole has been less exploited even if those organic molecules containing heterocyclic moiety are excellent as both cation and anion sensor signaling unit [49,50,51]. The acidic NH proton can interact with anion, whereas the nitrogen atom present in the ring can bind the cations [52]. The commercial availability of imidazole derivatives is a plus their use as chemosensors. Indeed, isoindole have been used in the development of drugs [53,54,55,56].

Herein, we report a fused isoindole imidazole fluorescent Schiff base chemosensor (1-{3-[(2-Diethylamino-ethylimino)-methyl]-2-hydroxy-5-methyl-phenyl}-2H-imidazo[5,1-a]isoindole-3,5-dione), which is labelled as **IIED**. Our hypothesis is based on the exploitation of an imidazole scaffold with Schiff bases, which are widely used in the field of metal ion detection [57,58,59,60,61,62,63,64]. The performed experimental and theoretical work demonstrated that **IIED** can detect Zn^2+^ with a limit of 0.073 μM. Aiming at maximizing the use of that new probe, its response in gel form was also assessed.

## 2. Results

### 2.1. Synthesis and Characterization

**Scheme 1:**
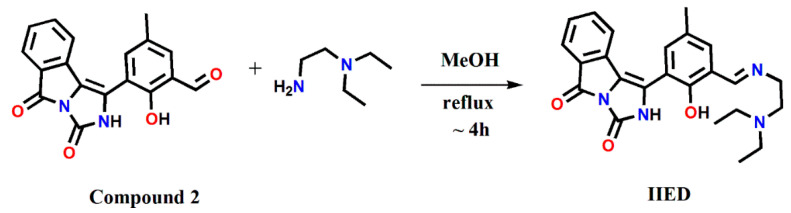
Synthetic procedure of **IIED**.

The probe **IIED (1-{3-[(2-Diethylamino-ethylimino)-methyl]-2-hydroxy-5-methyl-phenyl}-2*H*-imidazo[5,1-a]isoindole-3,5-dione)** was synthesized by condensing 3-(3,5-Dioxo-2,5-dihydro-3*H*-imidazo[5,1-a]isoindol-1-yl)-2-hydroxy-5-methyl-benzaldehyde (compound **2**) and *N*,*N*-Diethylethylenediamine. Compound **1** (1-(2-Hydroxy-5-methyl-phenyl)-2*H*-imidazo[5,1-a]isoindole-3,5-dione) was prepared from the standard procedure available elsewhere (Figure S1) [65]. Compound **2** was prepared by Duff reaction as previously reported by Chang et al. [66]. In the final step reaction, the Schiff base condensation product **IIED** was found in a powdery light orange form with a percentage of yield 57.8% (Scheme 1). The preparation of compound **1** and compound **2** were monitored by ^1^H NMR spectroscopic studies (Figures S1 and S2). The final step compound **IIED** were characterized by ^1^H NMR, ^13^C NMR, FT–IR, ESI–MS spectroscopic study (Figures S3–S6). FT-IR spectrum confirms the peak at 1642 cm^−1^ for the generation of imine bond (C=N). The ^1^H NMR peak at δ_H_ 8.53 is for aldehydic proton signal, δ_H_ 7.81–7.31 peaks are for the aromatic protons and and δ_H_ 3.6, 2.6, 2.5, 2.2, 0.95. peaks appeared for aliphatic protons. ESI-MS spectrum shows peak at 419.22 amu for [C_24_H_27_N_4_O_3_]^+^species. Further synthetic details can be found in Section 3 and in the Supporting Information.

### 2.2. Photophysical Studies of IIED towards Zn^2+^

**Figure 1:**
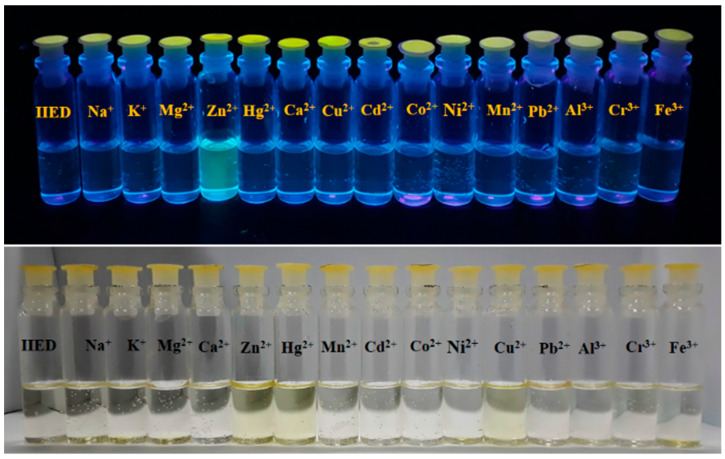
The changes under UV light (above)and day light (below)after addition of various cations to **IIED**.

The entire spectral behaviour was assessed in 3:7 HEPES buffer:DMSO medium (Figure 1). The UV–Visible spectra of the probe **IIED** (10^−5^ M) show peaks at 405 nm and 480 nm. The UV–Visible spectra of the probe in presence of various metal ions (Na^+^, K^+^, Ca^2+^, Mg^2+^, Hg^2+^, Ni^2+^, Fe^3+^, Cu^2+^, Co^2+^, Cd^2+^, Zn^2+^, Mn^2+^, Pb^2+^, Al^3+^, Cr^3+^) were recorded. The probe **IIED** changes its appearance from colorless to very pale yellow in the presence of Cu^2+^, Zn^2+^, Hg^2+^ ions a new peak at 467 nm had appeared for these three cations (Figure S8).

**Figure 2:**
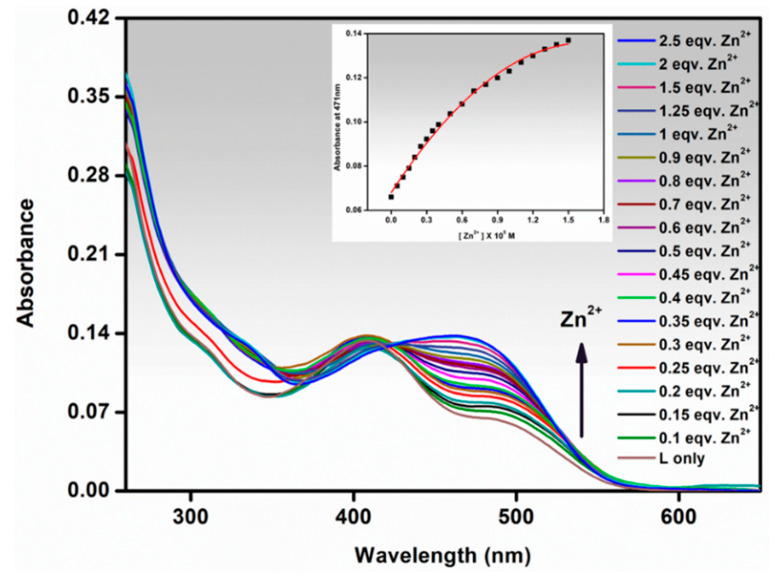
Absorbance titration spectra of **IIED** (10^−5^ M) in the presence of various concentrations of Zn^2+^ (up to 2.5 equiv.). Upward arrow indicates gradual increase of absorption at 467 nm with increasing concentration of Zn^2+^. [**Inset**: Absorbance of **IIED** vs. [Zn^2+^] plot at 467 nm].

The UV–Visible titration experiment of **IIED** was carried out for Zn^2+^ by the gradual addition of Zn^2+^ up to 2.5 µM. The absorbance peak at 467 nm gradually increased with the presence of isosbestic point at 419 nm which indicates that only one equilibrium is present between the **IIED** and **IIED** in presence of Zn^2+^ (Figure 2).

Benesi–Hildebrand (B–H) relation has been subsequently combined with the absorption titration spectral outputs according to Equation (1) [67]:

(1) $$\frac{1}{\left(A-A_{0}\right)}=\frac{1}{\left\{K\left(A_{\max}-A_{0}\right)\left[C\right]\right\}}+\frac{1}{\left(A_{\max}-A_{0}\right)}$$

where, A_0_ is the absorbance of ligand **IIED** only, A is the observed absorbance at that distinct wavelength in the presence of a particular concentration of the metal ion [C], A_max_ is the maximum absorbance value of the complex formed. K is the association constant (M^−1^) which was calculated from the slope of the linear plot and [C] is the concentration of the Zn^2+^ ion added during titration studies. The linear fit of the B–H plot of 1/(A − A_0_) vs. 1/[Zn^2+^] indicates 1:1 complex formation (Figure S12) between **IIED** and Zn^2+^ and association constant was found to be 1.2 × 10^4^ M^−1^ for absorbance. The stoichiometric ratio using absorbance value was further confirmed by Job’s plot analyses. The molar fraction of the ligand was shown the highest value 0.51, which also indicates 1:1 stoichiometry between Zn^2+^ and **IIED** (Figure S17). All these accumulated measures back up that the novel probe accommodates one Zn^2+^ ion only, an experimental finding that guide our molecular models.

A larger dissimilarity is observed for the fluorescence regime. We first confirmed that the emission spectra of the probe **IIED** (10^−6^ M) show no peak intensity (non–fluorescent) upon excitation at 480 nm. The emission spectra of the probe was subsequently measured in presence of all selected metal ions (Na^+^, K^+^, Ca^2+^, Mg^2+^, Hg^2+^, Ni^2+^, Fe^3+^, Cu^2+^, Co^2+^, Cd^2+^, Zn^2+^, Mn^2+^, Pb^2+^, Al^3+^, Cr^3+^). Emission signatures were recorded in 3:7 HEPES buffer solution:DMSO. Under UV light the probe shows dark to green emission upon addition of Zn^2+^. This optical response can be recognized by naked eye, a result that was not observed with any other ion. Such qualitative conclusion was confirmed by a quantitative analysis of the emission signatures. The probe **IIED** turns on fluorescence intensity at 558 nm (λ_ex_ = 480 nm) in the presence of Zn^2+^ ion only, among other cations mentioned above. It should be underlined that a complete competitive study in emission had been performed with IIED (Figures S9 and S10). The use of our probe with other metal ions demonstrated the selective output for Zn^2+^.

**Figure 3:**
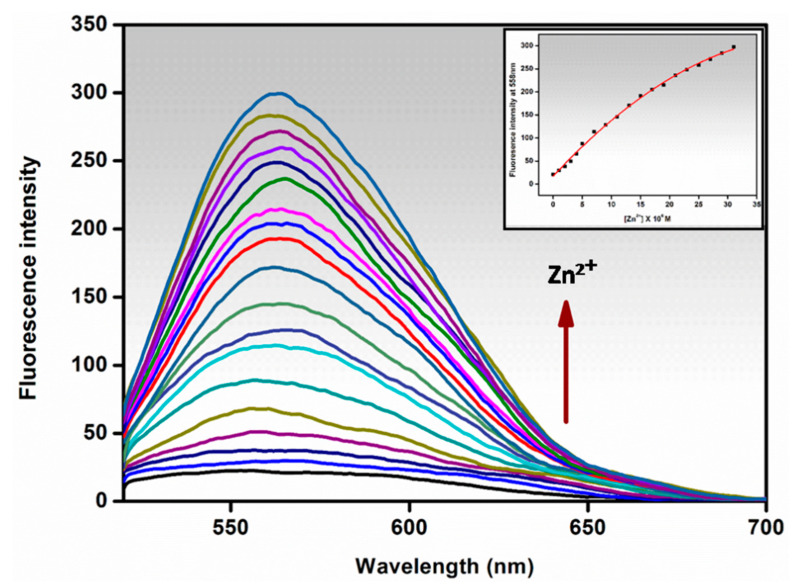
Emission titration spectra of **IIED** (10^−6^ M) in presence of various concentrations of Zn^2+^ (up to 25 equiv.). Upward arrow indicates gradual increase of emission at 558 nm with increasing concentration of Zn^2+^ ion (λ_ex_ = 480 nm). [**Inset:** Emission of **IIED** vs. [Zn^2+^] plot at 558 nm].

The fluorescence titration of **IIED** in presence of an increasing concentration of Zn^2+^ had been performed by the gradual addition of Zn^2+^ up to 25 µM in 3:7 HEPES buffer solution:DMSO. As illustrated in Figure 3, the 558 nm characteristic peak of the probe with Zn^2+^ directly correlates with Zn^2+^ concentration.

The emission spectral response towards **IIED** in presence of various counter anions as salts of Zn^2+^ (Zn(ClO_4_)_2_, ZnCl_2_, ZnBr_2_, Zn (NO_3_)_2_ and Zn(OAc)_2_) shows peaks at 558 nm, but the intensity varies slightly indicating negligible counter anion effect (Figure S11). This is a remarkable finding: **IIED** is proved to be versatile enough to reveal the presence of Zn^2+^ with no restriction on the parent-salt pair.

Consistently with absorption titration, emission titration spectral output was combined with a Benesi–Hildebrand (B–H) equation to find out the association constant (K) upon the complexation of **IIED** with Zn^2+^ ion according to Equation (2):

(2) $$\frac{1}{F_{X}-F_{0}}=\frac{1}{F_{\max}-F_{0}}+\frac{1}{K\left[C\right]}\left(\frac{1}{F_{\max}-F_{0}}\right)$$

where F_0_ is the emission of ligand **IIED** only, F_x_ is the emission of ligand at an intermediate Zn^2+^ concentration, and F_max_ is the maximum emission value of the complex formed. K is the binding constant and [C] is the concentration of Zn^2+^ ions. According to B–H expression, the measured emission [(F_max_ – F_0_)/(F_x_ – F_0_)] at 558 nm varied as a function of 1/[Zn^2+^] in a linear relationship, also indicated the formation of 1:1 stoichiometry between Zn^2+^and probe **IIED**. The association constant is found to be 3 × 10^4^ M^−1^ for emission (Figure S13), a results that is consistent with the predicted value by using absorbance, e.g., 1.2 × 10^4^ M^−1^ (see above).

The fluorescence quantum yield (Φ) was determined using quinine sulphate in 0.05 M H_2_SO_4_ as reference using the following Equation (3) [68]:

(3) $$\phi_{s}=\phi_{R}\frac{A_{s}}{A_{R}}\times \frac{{\mathrm{Abs}}_{R}}{{\mathrm{Abs}}_{S}}\times \frac{\eta_{S}^{2}}{\eta_{R}^{2}}$$

where A indicates the integrated area under the fluorescence curve, Abs stand for absorbance, η is the refractive index of the medium and Φ is the fluorescence quantum yield. Subscripts S and R stand for the respective specification for the studied sample and reference, respectively. The probe exhibits low fluorescence emission peak at 558 nm with quantum yield Φ = 0.036 upon excitation at 480 nm but in Zn^2+^ environment the quantum yield rises to Φ = 0.69.

The emission intensity of probe **IIED** in presence of Zn^2+^ was assessed at several pH values in the range of 4–10. In acidic region (pH < 6.0) there is no fluorescence enhancement in presence of Zn^2+^. On the contrary, in basic region (pH > 9.0) emission intensity gradually deceases. The effectivity of Zn^2+^ was observed in the range pH 7–8 and highest in pH 8. These findings suggest that the complexation phenomenon is optimal (i.e., the most stable) at the physiological pH region (Figure S14).

The limit of detection has been evaluated from the titration experiments for both absorption and emission study, using the Equation (4):

(4) $$\mathrm{DL}=K\times \frac{\sigma }{S}$$

where a threshold of K = 3 is imposed to ensure an acceptable signal-to-noise rate, and σ is the standard deviation of the blank solution and S is the slope of the calibration curve [69]. The calculated limit of detection for emission study of **IIED** with Zn^2+^ was 0.073 μM while for the absorption regime was 0.29 μm (Figures S15 and S16).

The fluorescence study is eventually completed by recording lifetime for **IIED** parent probe and its **IIED** + Zn^2+^ counterpart. For the records, these measure have been carried out in 3:7 HEPES buffer solution:DMSO at 480 nm, 298 K. Average fluorescence lifetimes (τ_avg_) were calculated from the decay times and pre–exponential factors using the following Equation (5):

(5) $$\tau_{\mathrm{avg}}=\frac{\sum \alpha_{i}\tau_{i}^{2}}{\sum \alpha_{i}\tau_{i}}$$

where α_i_ is the pre–exponential factor corresponding to the *i*th decay time constant, τ_i_.

**Figure 4:**
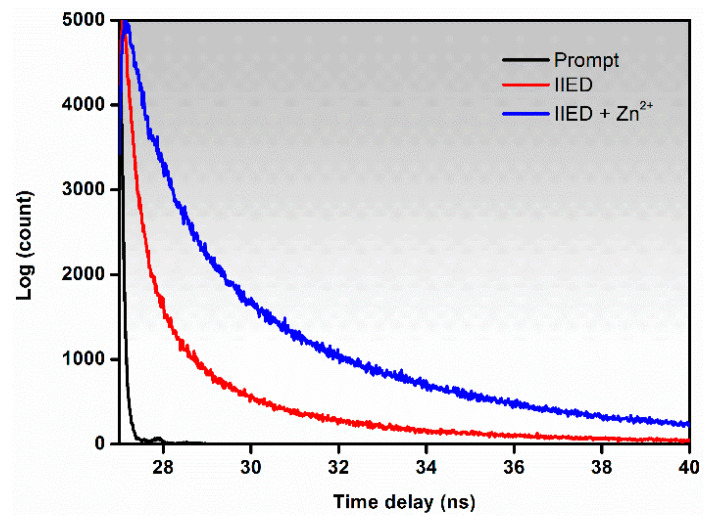
Time-resolved fluorescence decay of **IIED** in the absence and presence of Zn^2+^ in 3:7 HEPES buffer:DMSO solvent at 480 nm.

The radiative decay rate constant *k_r_* and the total nonradiative decay rate constant *k_nr_* of **IIED** and **IIED** + Zn^2+^ complex was calculated according to the equations *τ^−1^* *= k_r_ + k_nr_* and *k_r_ = Φ_f_/τ*. The fluorescence decay curves for **IIED** and its Zn^2+^ complex were fitted by three exponential functions. From the decay curve and fitting data, the average fluorescence lifetime (*τ*) of **IIED** and **IIED** + Zn^2+^ were estimated 2.74 ns and 3.65 ns. The values of all the fitting data were shown in Figure 4 and Table S1. The values of radiative and nonradiative decay rate constants for **IIED** indicating the nonradiative decay is the predominant process in the excited states which goes indeed reverse in the presence of Zn^2+^ where radiative decay is the predominant one, resulting in a strong fluorescence response.

### 2.3. Theoretical Calculations

**Figure 5:**
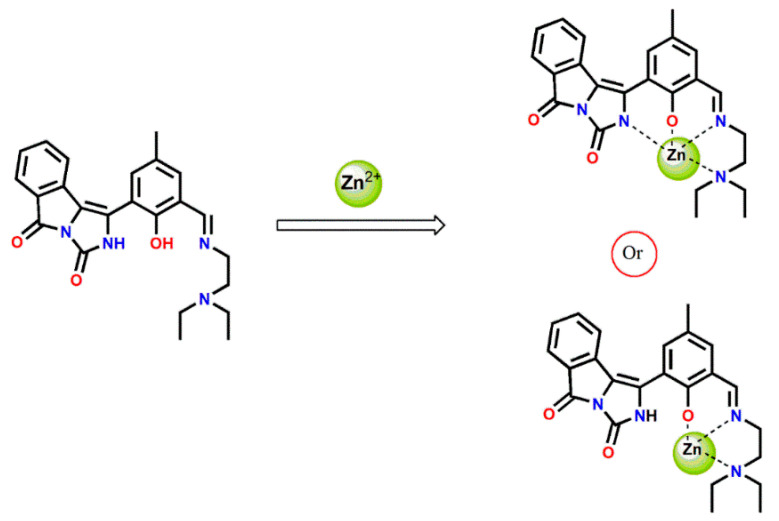
Left panel: parent **IIED** probe. Right panel: Complexes with Zn^2+^ as L(N) and L(NH) forms, displayed on the top and bottom, respectively.

As illustrated in Figure 5, two alternative structures become possible upon the Zn^2+^ coordination to **IIED**, labeled as L(N) and L(NH) according to their protonation state. Despite all accumulated experimental evidence, the participation of -NH proton in the binding of Zn^2+^ is not confirmed by the experimental optical signatures.

**Figure 6:**
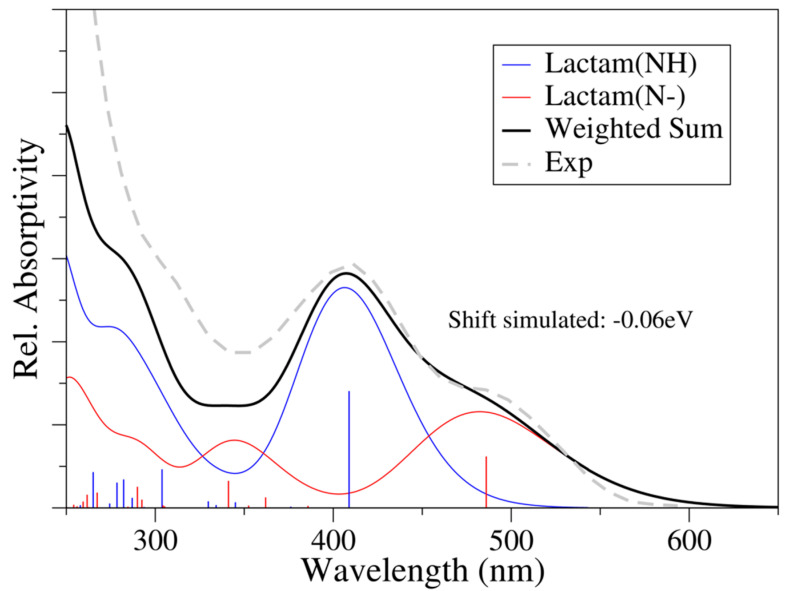
Spectra simulated for the ligand in lactam form, both neutral and N-deprotonated. Stick transitions computed with TD—PBE0 were convoluted with Gaussian functions with HWHM = 0.25 eV. The spectra simulated for the N—deprotonated form was scaled by 0.5 to match the experimental spectra (shown as dashed lines).

Density functional theory (DFT) and time dependent-DFT (TD-DFT) calculations were performed to determine the main form responsible of the measured photophysical activity. All structures were first fully optimized without imposing any symmetry restriction. We considered both the neutral (LN-) and the N-deprotonated (LNH) species for the free ligand. The computed pKa was in the range of 7.2–7.4. Such a value is significantly below the expected range for lactams (pKa = 11–18), which can be explained by the stabilization of the N- site by a hydrogen bond with the phenol group. This hypothesis was confirmed by analyzing a conformer where the hydrogen bond formation was impossible, leading to a pKa of ~15. We further checked that the O-deprotonated compound was less stable in DMSO solution than the N-deprotonated one. The spectra of both the neutral and deprotonated ligands were computed using the optimized structures in the gas phase (Figure 6). A phenomenological broadening of 0.25 eV was applied to the 30 computed transitions.

Our simulations indicate that both the neutral and deprotonated forms give rise to the experimental spectrum. Concretely, the deprotonated form leads to a low energy band, ~0.4 eV (~70 nm) below the neutral form, and it is consistent with the shoulder observed in the experimental spectrum around 500 nm. Indeed, a semi-quantitative agreement is achieved by combining both spectra with the deprotonated form scaled by 0.5. This scaling corresponds to a dissociation fraction of 0.333. Considering that the pH is buffered at 7.4, such a ratio would correspond to a pKa = 7.7, which agrees very well with our theoretical estimation.

**Figure 7:**
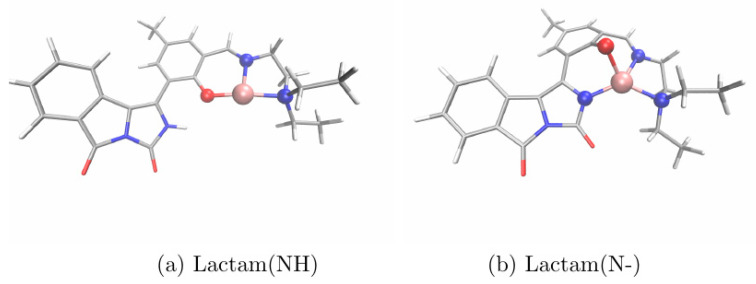
Optimized structures of Zn-Ligand. The amide site is considered protonated (LNH, left (**a**)) or deprotonated (LN-, right (**b**)).

We next turned to the complexes, considering in all cases that Zn binds to the phenol oxygen, replacing the hydrogen. In contrast, the amide site is considered both protonated (LNH) and deprotonated (LN-). The latter lead to a tetradentate chelate, while LNH binds Zn cation by three sites, as shown in Figure 7.

**Figure 8:**
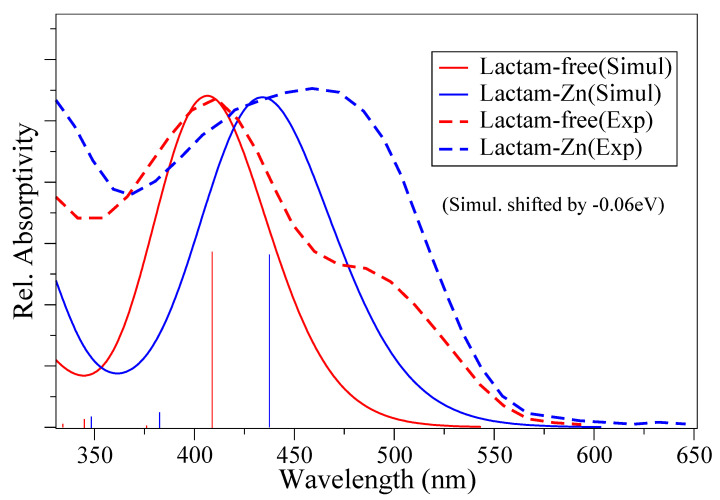
Experimental and computed absorption spectra of the two possible compounds upon Zn^2+^ complexation, e.g., L(N) and L (NH), displayed with blue and red lines, respectively.

The spectra for the free and complex compounds with N-protonated sites is shown in Figure 8. The complex shows a large pKa (>15), typical for lactams; the contribution of the N-deprotonated complex is consequently not expected. Although the predicted shift is smaller than the observed signatures, which is the consequence of introducing the metal Zn into the model system, our simulations correctly mimic a red-shift of the complex compared to the free ligand, agreeing with the experimental observation.

### 2.4. Sensing Mechanism

The absorption profile of Compound **1** was originally characterized by Ray et al., which exhibits two absorption peaks at 320 nm and 395 nm [70]. **IIED** is a modified version of Compound **1** where we introduced an imine (-C=N) moiety and also increasing the conjugation. As a consequence, these absorption peaks are red shifted at 405 nm and 480 nm in the novel **IIED** probe. That trend is correctly reproduced by our TD-DFT calculations. As discussed above, the **IIED** showed no emission band in 3:7 HEPES buffer:DMSO solution upon excitation at 480 nm while fluorescence is switched on after Zn^2+^ coordination. That dissimilarity in the emission arises from a ESIPT (Excited State Intramolecular Proton Transfer) process. Indeed, earlier works demonstrated that probes with hydroxy-imine moieties are associated to lower fluorescence because of ESIPT equilibria [71]. Such scenario is correctly reproduced by our computational approach, which shows that the spectroscopic transition is characterized by a partial transfer of electron density from the phenolic oxygen to the imine nitrogen, as concluded from the inspection of the molecular orbitals involved (Figure S19). That electronic rearrangement activates the ESIPT and consequently reduces fluorescence. The substitution of the proton by the metal in the **IIED** + Zn^2+^ complex cancels the ESIPT and results in the chelation enhanced fluorescence (CHEF) phenomena at 558 nm.

### 2.5. Reversibility and Application in Gel Phase

Our main goal is to report a probe with application in real scenarios. A critical prerequisite for optimizing the use of **IEED**, which was performed by using sodium salt of ethylenediaminetetraacetic acid (Na_2_EDTA) solution in emission spectra. In the presence of EDTA, the 558 nm peak of the **IIED** + Zn^2+^ complex had been quenched and reappear upon addition of Zn^2+^ solution again (Figure S18). The process has been repeated for few cycles, indicating a reversible coordination between **IIED** and Zn^2+^.

**Figure 9:**
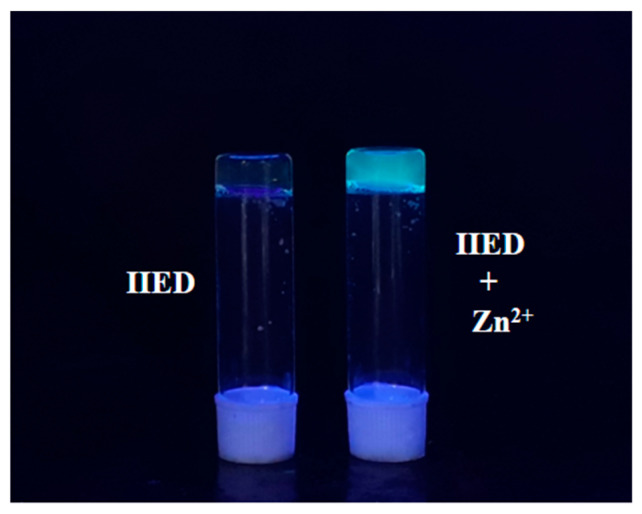
Visual change of **IIED** in the presence of Zn^2+^ in gel form under UV light.

Finally, we have also checked the stability of our probe in gel medium. The gel has been prepared as the method described in literature using Poloxamer 407 [72,73]. In this gel phase also the probe **IIED** shows change from dark to green fluorescence under UV light in presence of Zn^2+^ and the naked eye output is shown in Figure 9.

## 3. Materials and Methods

### 3.1. Materials and Instruments

Ninhydrin, p–cresol, HMTA (Hexamethylenetetramine), TFA (Trifluoroacetic acid), N, N-Diethylethylenediamine was purchased from Sigma–Aldrich, Kolkata, India, urea purchased from Merck, Kolkata, India and all were used as received. The salts of the cations were also purchased from Sigma Aldrich, Kolkata, India. Solvents for the syntheses were purchased from commercial sources and used as received. ^1^H and ^13^C NMR spectrum were recorded in DMSO-*d*_6_ with TMS as internal standard on a Bruker, AV300 Supercon Digital NMR system with dual probe. The FT–IR spectra were recorded from KBr pellets in the range of 400–4000 cm^−1^ on a Perkin-Elmer Spectrum 100 spectrometer. Elemental analyses for C, H and N were performed on a Perkin–Elmer 2400 II analysers. The ESI-MS experiments were performed on Waters Xevo G2-S QTOF mass spectrometer. The absorption and emission spectral studies were performed on a Hitachi UV–Vis U–3501 spectrophotometer and a Perkin–Elmer LS55 fluorimeter, respectively. Time-resolved fluorescence lifetime measurement were performed in Horiba Jobin Yvon Fluorocube-01-NLtime-correlated single photon counting (TCSPC) set up with picosecond delta diode (DD–375L) operating at λ_ex_ = 480 nm and a repetition rate of 1 MHz as excitation source.

### 3.2. Computational Details

Computational methods were implemented to predict the absorption spectra of both the new **IIED** probe as well as two the possible complexes after Zn^2+^ complexation, which were labelled as L(N) and L(NH) depending on their protonation state. These calculations were conducted in the framework of the density functional theory (DFT) and the time dependent (TD-DFT) schemes by using the PCM-PBE0/cc-pVTZ level of theory [74,75]. For the records, the addition of a basis with pseudopotentials was also assessed. The use of LANL2TZ+ on the Zn^2+^ center has a minor effect on the structures, e.g., predicts the same chelation as that not using pseudopotentials. The nature of all located structures was confirmed as real minima (stable) forms by analyzing the associated vibrational modes. The absence of imaginary frequencies confirm that all optimized structures are real minima in the potential energy surface rather than saddle points. Geometries were eventually used to assess the vertical transition energies used for predicting the absorption spectra of these species. The pK_a_ values were evaluated at two levels of theory. Our first choice is based on the protocol described by Rossini et al. [76], computing frequencies at B3LYP/6-31G(d,p) and single point energies with PCM at B3LYP/cc-pVQZ [77]. For the free energy of the proton in gas phase a value of 6.28 kcal/mol was used, while for the solvation free energy of the proton in DMSO, we adopted the theoretical estimation provided by Rossini et al., −266.4 kcal/mol [76]. The empirical pK_a_ prediction module by Schrödinger is also used as a second theoretical framework [78,79,80]. These schemes yield to very similar pKa values, 7.2 and 7.4, respectively. All DFT and TD-DFT calculations were performed with Gaussian 16 [81].

### 3.3. Synthesis of the Ligand

The **IIED** synthesized in three simple steps. The 1-(2-Hydroxy-5-methyl-phenyl) -2*H*-imidazo[5,1-a]isoindole-3,5-dione (Compound **1**) was prepared by the procedure found in literature [65]. The 3-(3,5-Dioxo-2,5-dihydro-3*H*-imidazo[5,1-a]isoindol-1-yl)-2-hydroxy-5-methyl-benzaldehyde (Compound **2**) was prepared by Duff reaction mechanism by HMTA and TFA found in literature [66]. In methanolic solution of Compound **2** (160 mg, 0.5 mmol), *N*,*N*-Diethylethylenediamine (72 µL, 0.5 mmol) was added dropwise and the color changes from yellow to orange. Then the solution was refluxed for 4 hrs and light orange colored precipitate was filtered through suction filtration and air dried. yield = 57.8% (120 mg, 0.29 mmol). Anal. calc. for C_24_H_26_N_4_O_3_: C, 68.88; H, 6.26; N, 13.39; Found: C, 68.7; H, 6.24; N, 13.4; ^1^H NMR (300 MHz, DMSO-*d*_6,_ 290 K, TMS) δ_H_(ppm): 8.53 (s, 1 H), 7.81−7.59 (m, 2 H), 7.45−7.31 (m, 4 H), 3.6(t, 2 H), 3.51 (s, −NH), 2.6 (t, 2 H), 2.52(q, 4 H), 2.27(s, 3 H), 0.95(m, 6 H); ^13^C NMR (75 MHz, DMSO–d_6_) δ_C_: 160.58, 158.79, 156.03, 142.37, 129.38, 128.97, 128.48, 127.47, 126.52, 122.62, 121.67, 120.97, 117.65, 115.63, 113.00, 111.85, 111.82, 49.88, 48.14, 42.66, 26.18, 24.95, 15.58, 7.24; FT–IR (KBr, cm^−1^):3518, 3237, 2966, 1762, 1682, 1642, 1607, 1441, ESI-MS (*m*/*z*), ion Calculated: 418.20 amu; Found: 419.22 amu [**IIED +** H^+^].

### 3.4. Sample Preparation for Spectroscopic Studies

Spectroscopy graded solvents were used for preparing all the stock and working solutions. Spectroscopy graded DMSO were used for the stock solution preparation and mixture of DMSO with HEPES buffer (pH = 7.4, 25 mM) was used in the spectral studies. The absorption and emission experiments of probe **IIED** was carried out in HEPES buffer: DMSO (3:7, *v*/*v*) mixture. All experiments were carried out at room temperature of 298 K. The stock solutions of the cations were prepared by the perchlorate salts. In UV–Visible and fluorometric experiment, a stock solution of 10^−3^ (M) and 10^−4^ (M) **IIED** was filled in a quartz optical cell of 1.0 cm optical path length to achieve a final concentration of the solution of **IIED** (10^−5^ (M) and 10^−6^ (M)) in 2000 µL. After 1 min, each spectral data was recorded with the addition of cation solution by using micropipette.

## 4. Conclusions

In this paper, we have designed, and synthesized an isoindole-imidazole skeleton based chemosensor that is highly selective for Zn^2+^ in 3:7 HEPES buffer: DMSO medium. The probe itself shows a weak fluorescence intensity (quantum yield, 0.036), and the addition of zinc ion enhanced the fluorescence intensity 19-fold (quantum yield, 0.69). The competitive study confirms high selectivity for Zn^2+^ against other common cations. The detection limit for zinc ion was 0.073 μm, which is significantly lower than the WHO guideline (76.5 mM). Addition of EDTA to the probe: Zn^2+^ ensemble quenched the fluorescence, indicating the reversibility of Zn^2+^ binding. Zn^2+^ titrations and Job’s plot analysis indicated the formation of a 1:1 probe: Zn^2+^ complex with a binding constant of 3 × 10^4^ M^−1^. The sensing application has been expanded successfully to gel form also. DFT/TD-DFT calculations also pointed towards the sensing mechanism along with the probable binding mode. If low concentrations of Zn^2+^ are required, our recommendation is to exploit the emission ability of the **IIED** probe.

## Data Availability

The data presented in this study are available upon request from the corresponding author.

## Supplementary Materials

The following supporting information can be downloaded at: https://www.mdpi.com/article/10.3390/molecules27092859/s1, ^1^H, ^13^C NMR, ESI-MS, FT-IR spectrum of probe IIED, spectrophotometric study, frontier molecular orbitals.

## Sample Availability

Samples of the compounds are available from the authors.
