# Supplementary material for: Turn on Fluorescence Sensing of Zn2+ Based on Fused Isoindole-Imidazole Scaffold
Source: Molecules. 2022 Apr 30;27(9):2859. doi: 10.3390/molecules27092859 (PMC9103770; doi:10.3390/molecules27092859)
Supplement: Supplementary file 1 [file molecules-27-02859-s001.zip › molecules-1629302-supplementary.pdf]

<u>*Supplementary Materials*</u>

Turn on Fluorescence Sensing of Zn^{2+} Based on Fused Isoindole-Imidazole Scaffold

Sutapa Sahu [1], Yeasin Sikdar [2], Riya Bag [1], Javier Cerezo [3], José P. Cerón-Carrasco [4,*] and Sanchita Goswami [1,*]

1 Department of Chemistry, University of Calcutta, 92, A.P.C. Road, Kolkata 700009, India; sutapa.sahu11@gmail.com (S.S.); riyabag.chem@gmail.com (R.B.)
2 Department of Chemistry, The Bhawanipur Education Society College, 5, LalaLajpat Rai Sarani, Kolkata 700020, India; y.sikdar@gmail.com
3 Departamento de Química, Universidad Autónoma de Madrid, 28049 Madrid, Spain; javier.cerezo@uam.es
4 Centro Universitario de la Defensa, Academia General del Aire, Universidad Politécnica de Cartagena, C/Coronel López Peña S/N, Santiago de La Ribera, 30720 Murcia, Spain
* Correspondence: jose.ceron@cud.upct.es (J.P.C.-C.); sgchem@caluniv.ac.in (S.G.)

Contents of the Supporting Information

	Page No.
**Figure S1.** ^{1}H NMR spectrum of **compound 1** in *d6*–DMSO.	3
**Figure S2.** ^{1}H NMR spectrum of **compound 2** in *d6*–DMSO.	3
**Figure S3.** ^{1}H NMR spectrum of **IIED** in *d6*–DMSO.	4
**Figure S4.** ^{13}C NMR spectrum of **IIED** in *d6*–DMSO.	4
**Figure S5.** ESI–MS spectrum of **IIED.**	5
**Figure S6.** FT–IR spectrum of **IIED.**	5
**Figure S7.** ESI–MS spectrum of **IIED+** Zn^{2+}complex.	6
**Figure S8.** Absorbance spectra of **IIED** (10^{-5} M) in presence of various cations (Na^{+}, K^{+}, Ca^{2+}, Mg^{2+}, Hg^{2+}, Ni^{2+}, Fe^{3+}, Cu^{2+}, Co^{2+}, Cd^{2+}, Zn^{2+}, Mn^{2+},	6

Pb^{2+}, Al^{3+}, Cr^{3+}) in (3:7 v/v)HEPES buffer : DMSO medium.	
**Figure S9.** Emission spectra of **IIED** (10^{-6} M) in presence of various cations (Na^+, K^+, Ca^{2+}, Mg^{2+}, Hg^{2+}, Ni^{2+}, Fe^{3+}, Cu^{2+}, Co^{2+}, Cd^{2+}, Zn^{2+}, Mn^{2+}, Pb^{2+}, Al^{3+}, Cr^{3+}) in (3:7 v/v)HEPES buffer : DMSO medium.	7
**Figure S10.** Emission intensity of **IIED** (10^{-6} M) in the presence of 25 equiv. of different cations in absence of same equiv. of Zn^{2+} in solution [the blue bar portion]. Emission intensity of a mixture of **IIED** with 25 equiv. of other cations followed by addition of same equiv. of Zn^{2+} to the solution (λ_{em}=558 nm) [the orange blue bar portion].	7
**Figure S11.** Emission intensity of **IIED** in present different anion of Zn^{2+} of salts at 558 nm.	8
**Figure S12.** Benesi-Hildebrand plot for determination of binding constant of **IIED** with Zn^{2+} for absorbance.	8
**Figure S13.** Benesi-Hildebrand plot for determination of binding constant of **IIED** with Zn^{2+} for emission.	9
**Figure S14.** Emission of **IIED** in absence and in presence of Zn^{2+} at different pH values at 558 nm.	9
**Figure S15.** Determination of the detection limit of **IIED** in presence of Zn^{2+} for absorbance at 467 nm.	10
**Figure S16.** Determination of the detection limit of **IIED** in presence of Zn^{2+} for emission at 558 nm.	10
**Figure S17.** Job's plot for the identification of stoichiometry using absorbance values.	11
**Figure S18.** (A) The reversible colorimetric switch by alternate addition	11

of $Zn^{2+}$and EDTA in (3:7, v/v) HEPES buffer:DMSO medium. (B) Emission spectrum of **IIED** shows reversibility towards Zn^{2+} in presence of EDTA in (3:7 v/v) HEPES buffer : DMSO medium. (C) Visual change of **IIED** under UV light in the presence of Zn^{2+} and EDTA.	
**Table S1.** Time resolved fluorescence data	12

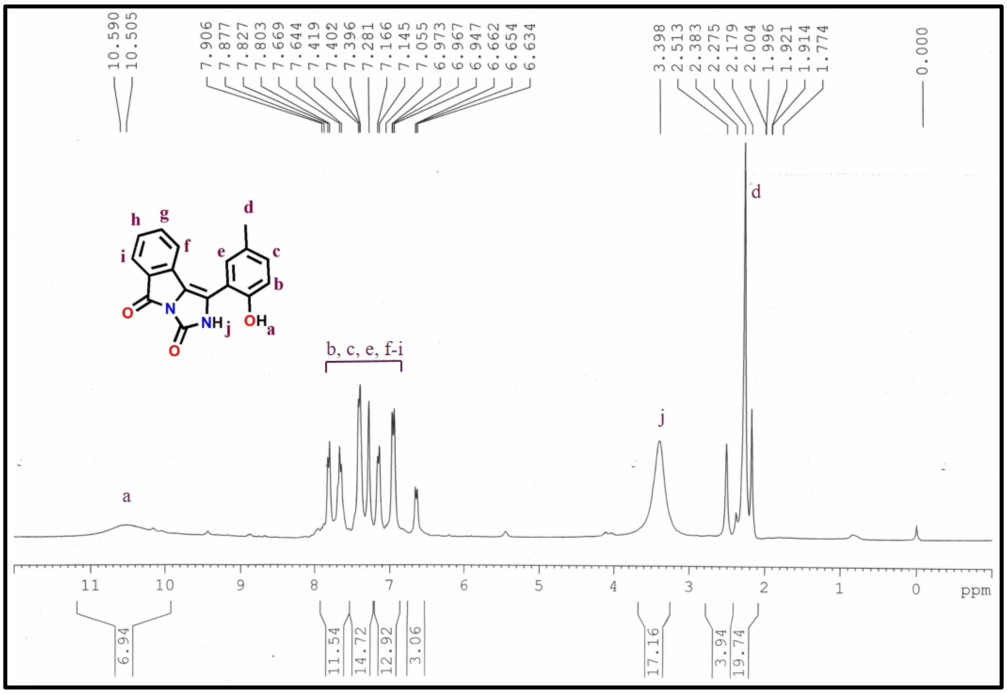

Figure S1.¹H NMR spectrum of **compound 1** in *d₆*–DMSO.

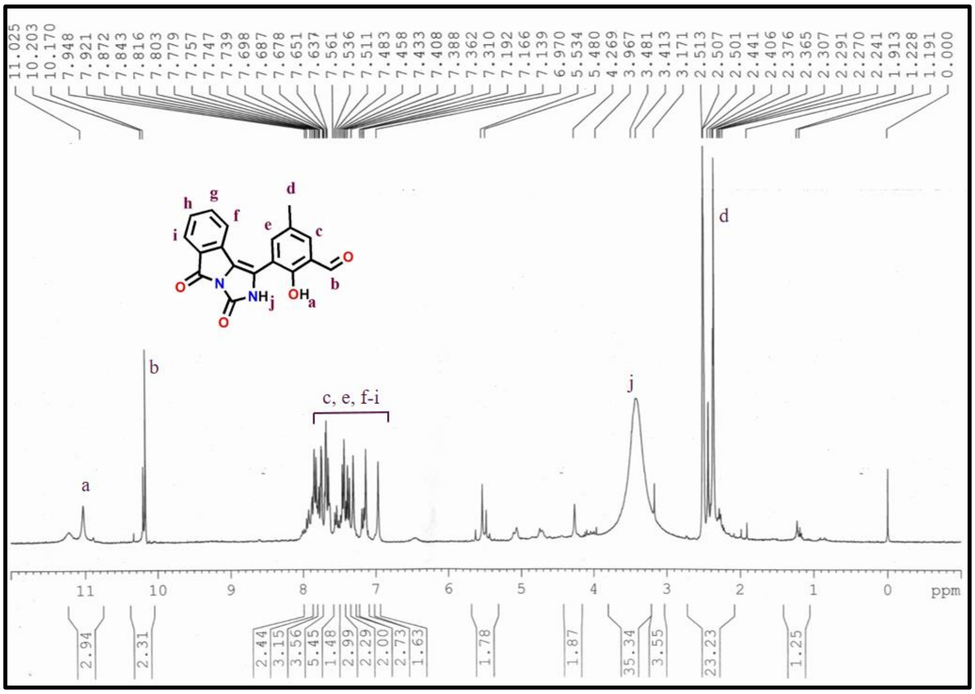

Figure S2.^{1}H NMR spectrum of **compound 2** in d_6–DMSO.

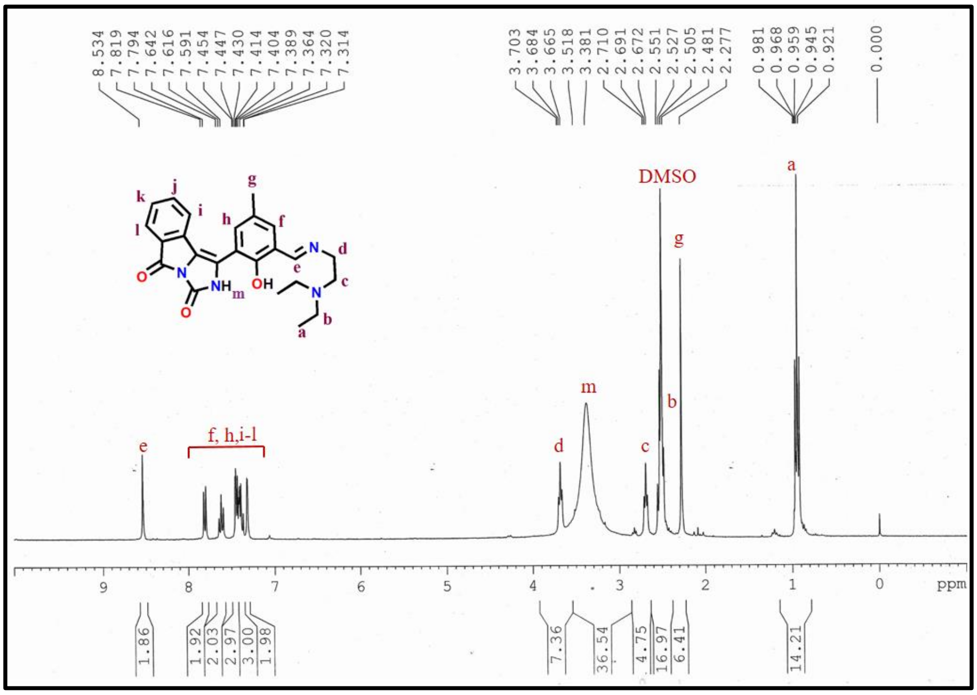

Figure S3.^{1}H NMR spectrum of **IIED** in d_6–DMSO.

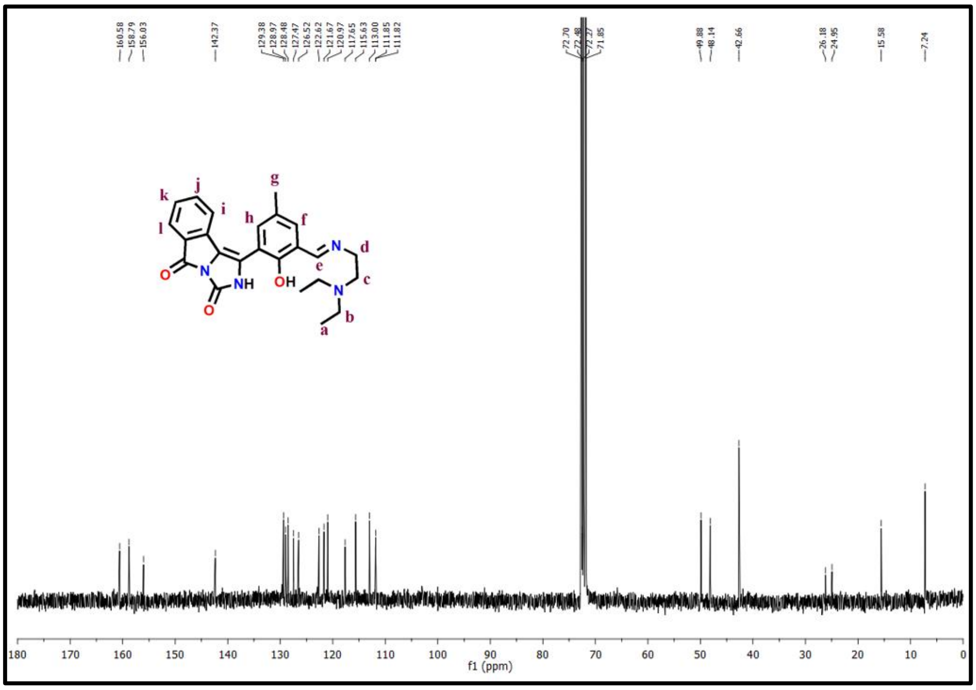

Figure S4. ^{13}C NMR spectrum of **IIED** in d_6–DMSO.

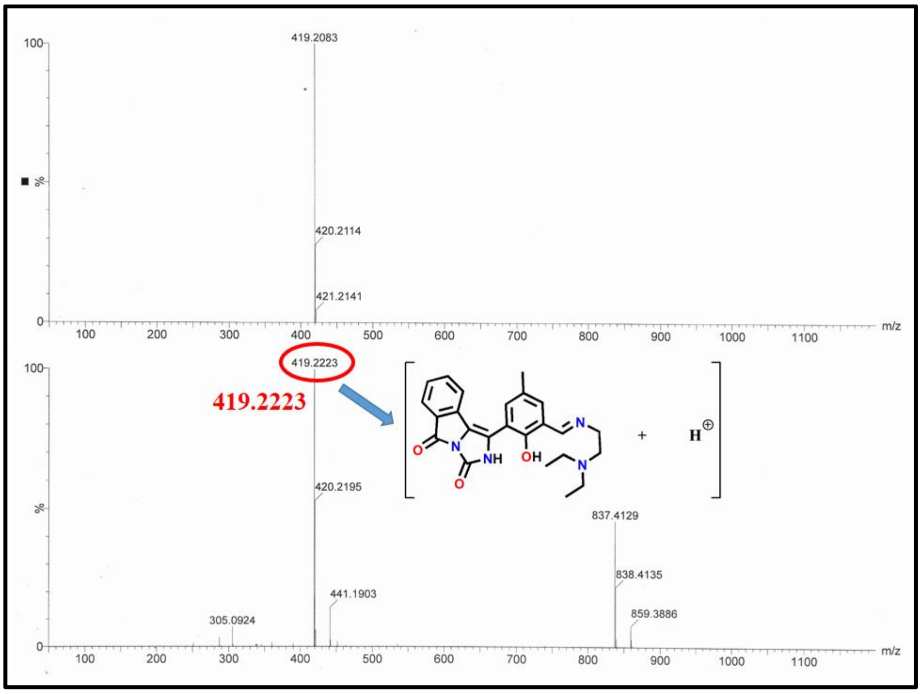

Figure S5. ESI–MS spectrum of **IIED.**

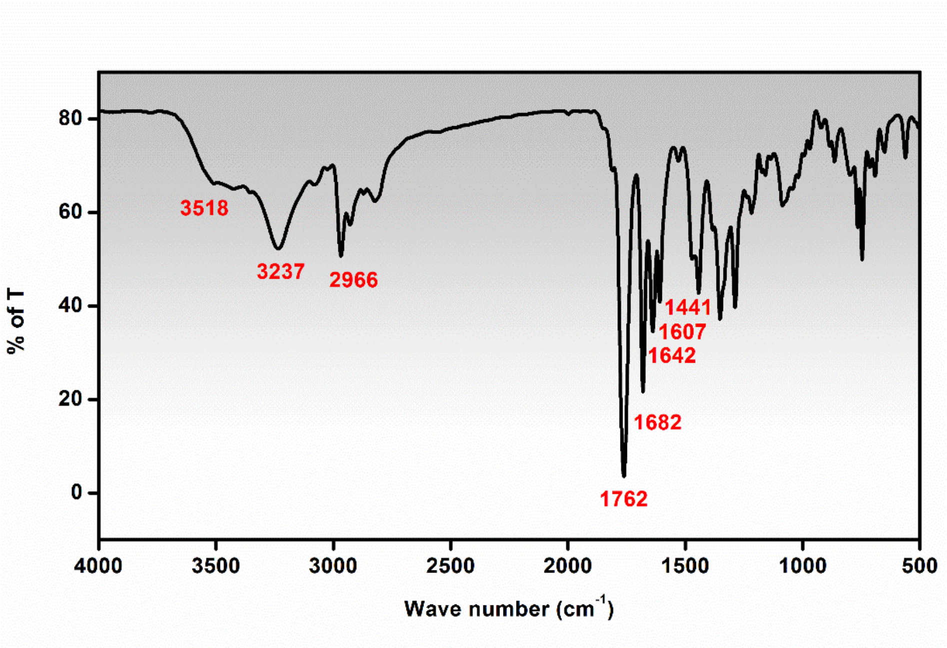

Figure S6. FT–IR Spectrum of **IIED.**

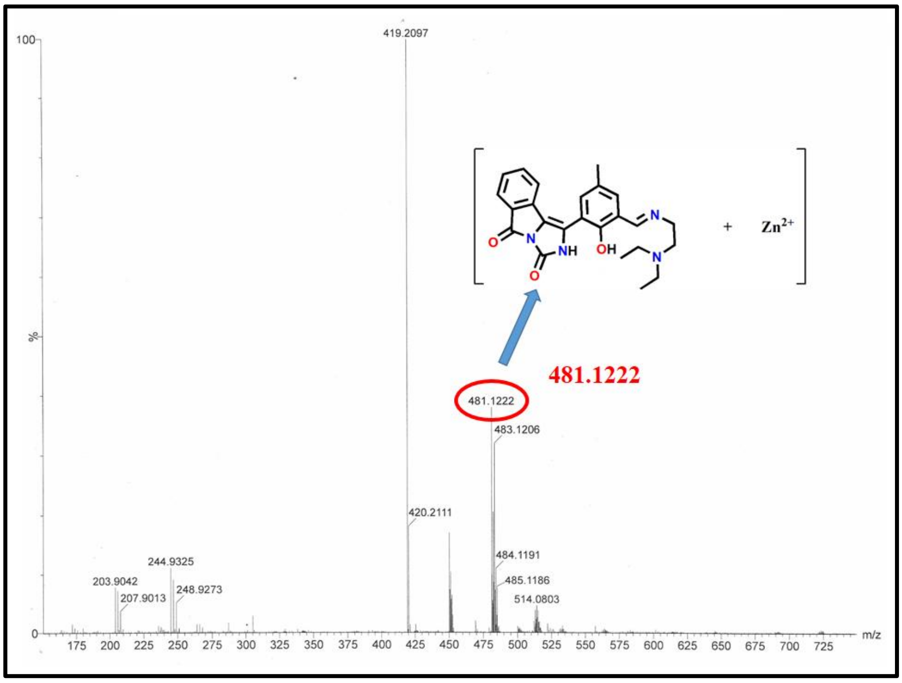

Figure S7. ESI–MS spectrum of **IIED+** Zn^{2+}complex.

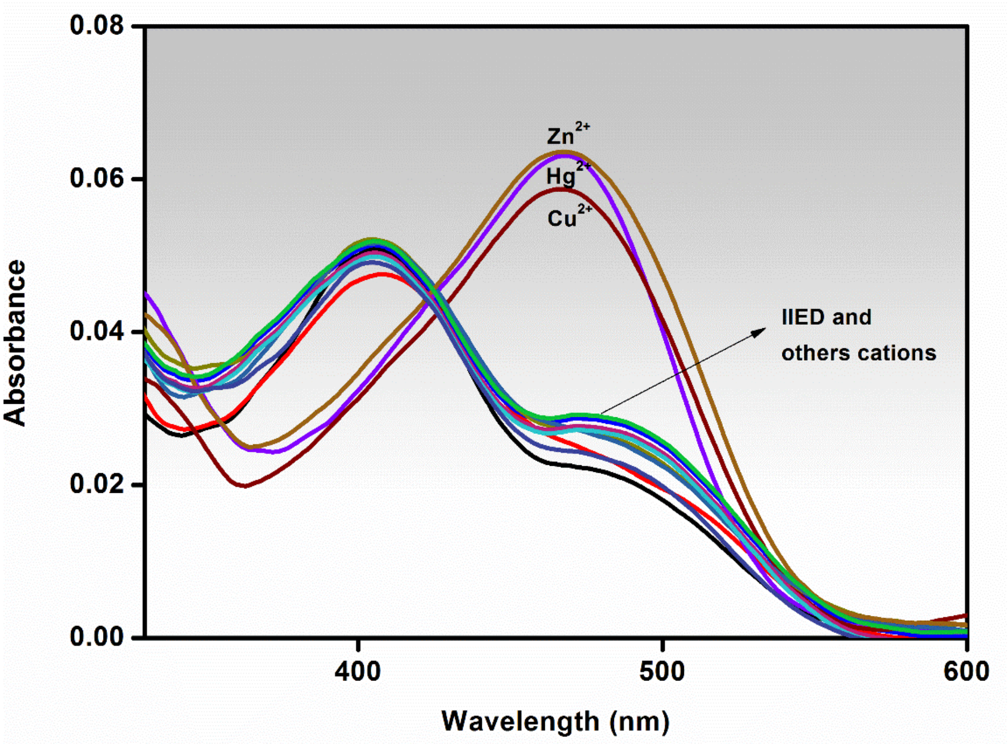

Figure S8. Absorbance spectra of **IIED** (10^{-5} M) in presence of various cations (Na^+, K^+, Ca^{2+}, Mg^{2+}, Hg^{2+}, Ni^{2+}, Fe^{3+}, Cu^{2+}, Co^{2+}, Cd^{2+}, Zn^{2+}, Mn^{2+}, Pb^{2+}, Al^{3+}, Cr^{3+}) in (3:7 v/v)HEPES buffer : DMSO medium.

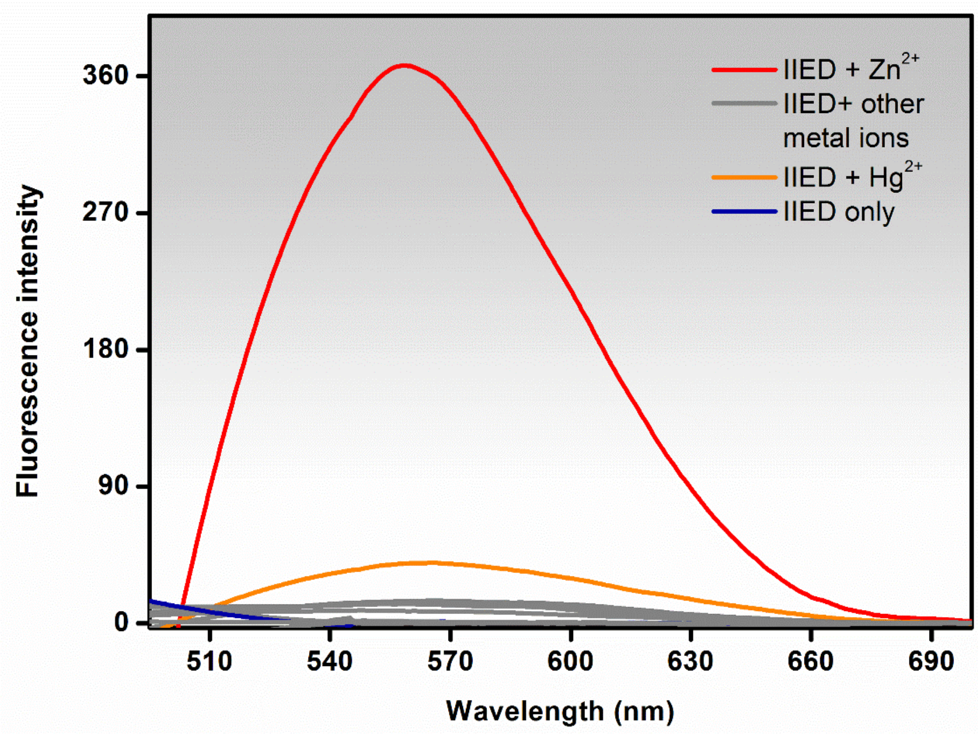

Figure S9. Emission spectra of **IIED** (10^{-6} M) in presence of various cations (Na^+, K^+, Ca^{2+}, Mg^{2+}, Hg^{2+}, Ni^{2+}, Fe^{3+}, Cu^{2+}, Co^{2+}, Cd^{2+}, Zn^{2+}, Mn^{2+}, Pb^{2+}, Al^{3+}, Cr^{3+}) in (3:7 v/v)HEPES buffer : DMSO

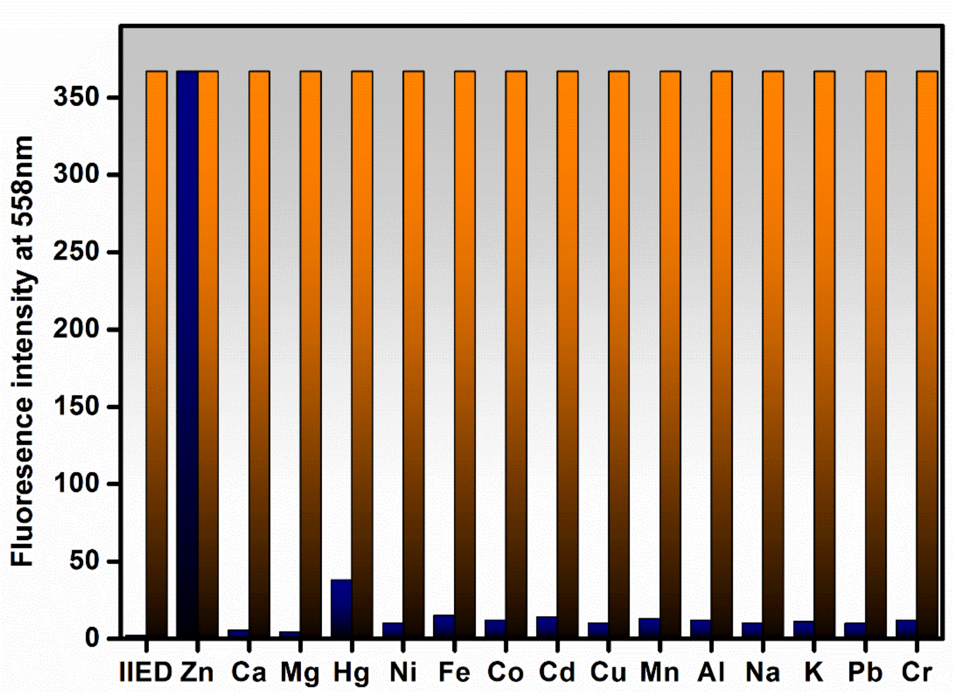

Figure S10. Emission intensity of **IIED** (10^{-6} M) in the presence of 25 equiv. of different cations in absence of same equiv. of Zn^{2+} in solution [the blue bar portion]. Emission intensity of a mixture of **IIED** with 25 equiv. of other cations followed by addition of same equiv. of Zn^{2+} to the solution (λ_{em}=558 nm) [the orange blue bar portion].

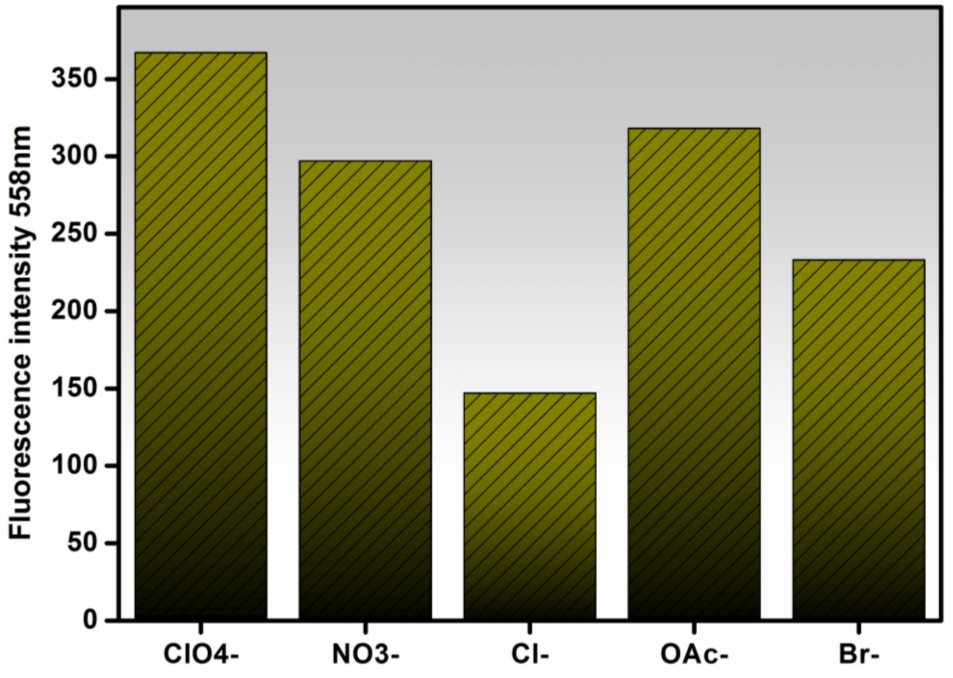

Figure S11. Emission intensity of **IIED** in present diiferent anion of Zn^{2+} of salts at 558 nm.

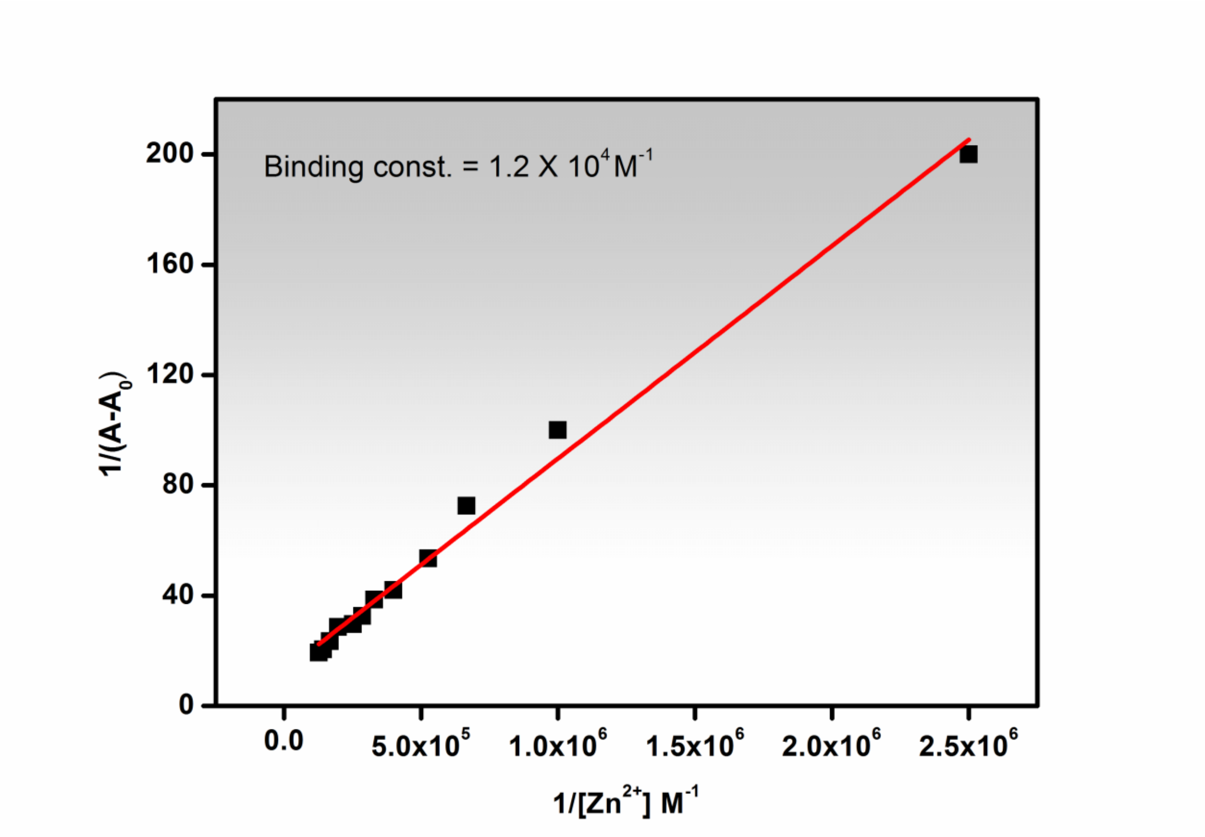

Figure S12. Benesi-Hildebrand plot for determination of binding constant of **IIED** with Zn^{2+} for absorbance.

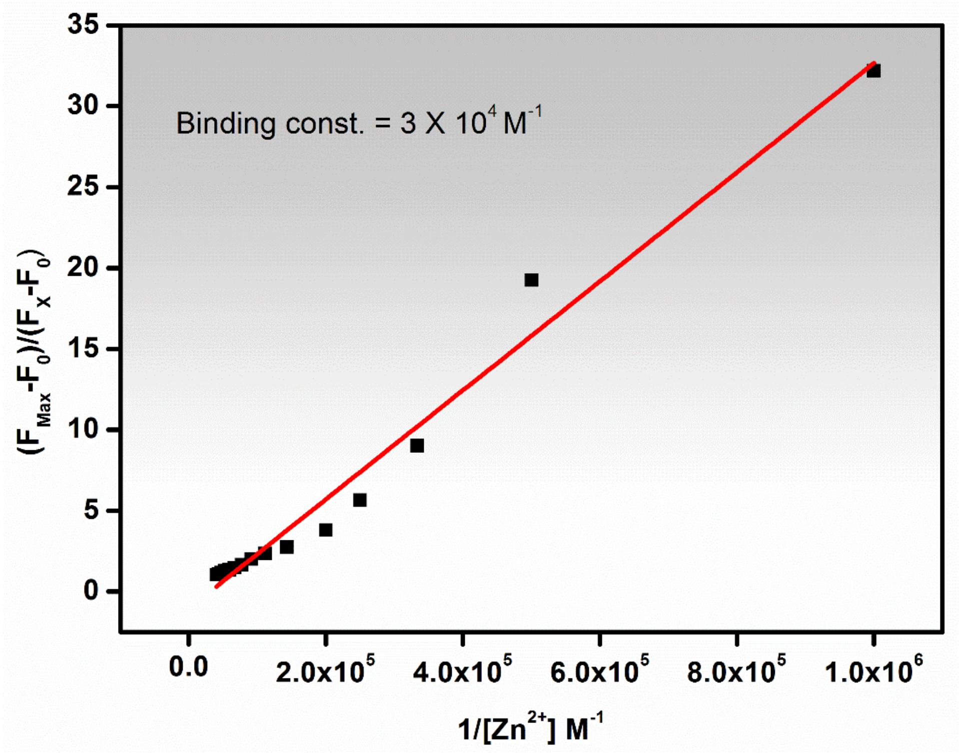

Figure S13. Benesi-Hildebrand plot for determination of binding constant of **IIED** with Zn^{2+} for emission.

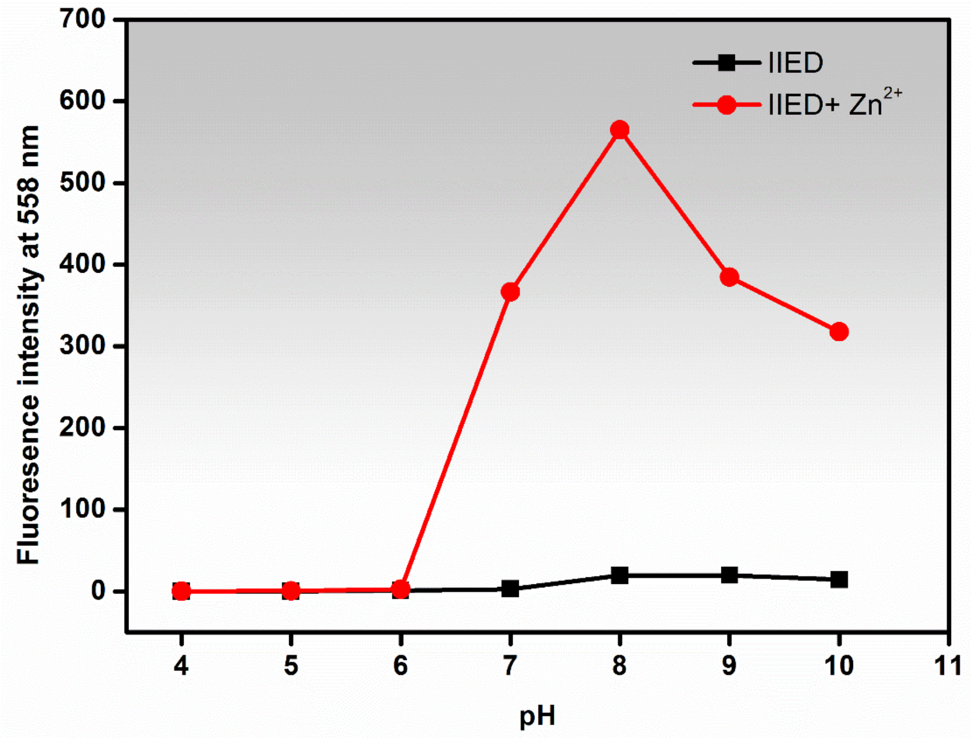

Figure S14. Emission of **IIED** in absence and in presence of Zn^{2+} at different pH values at 558 nm.

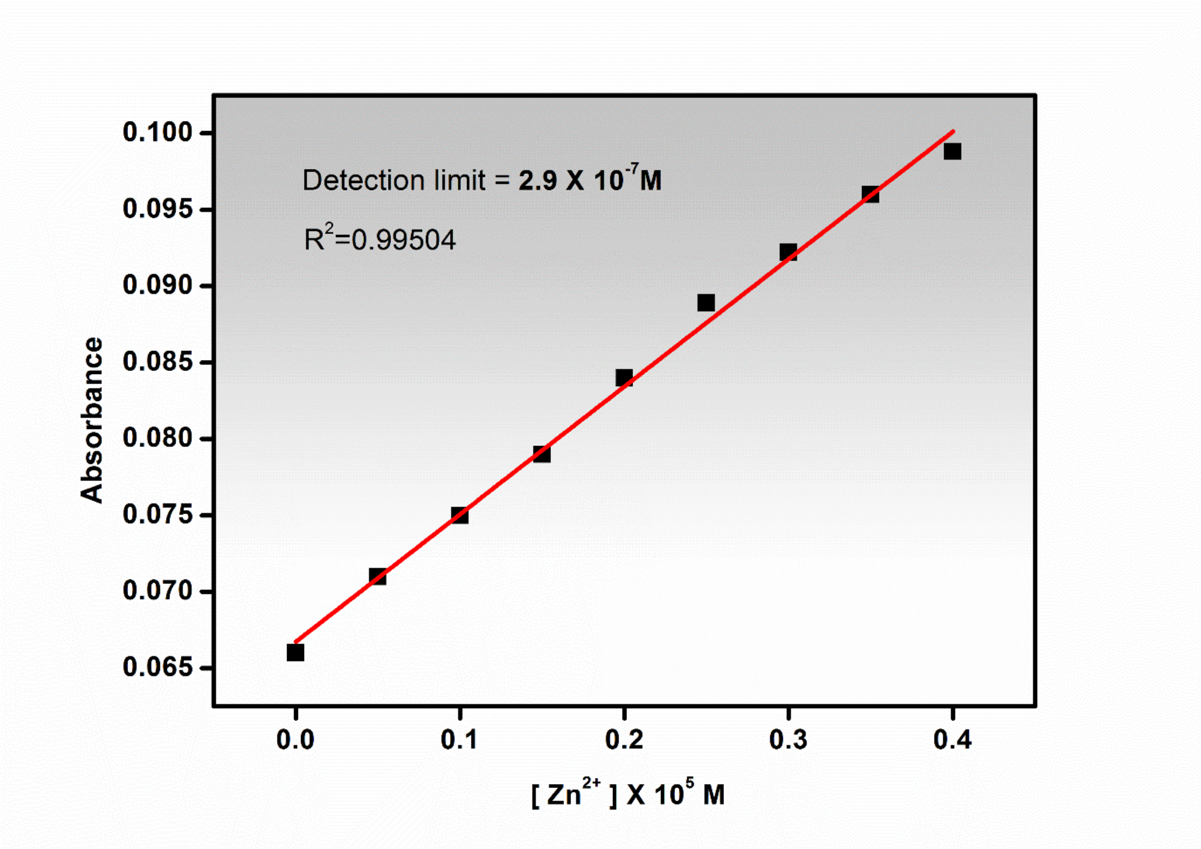

Figure S15. Determination of the detection limit of **IIED** in presence of Zn^{2+} for absorbance at 467 nm.

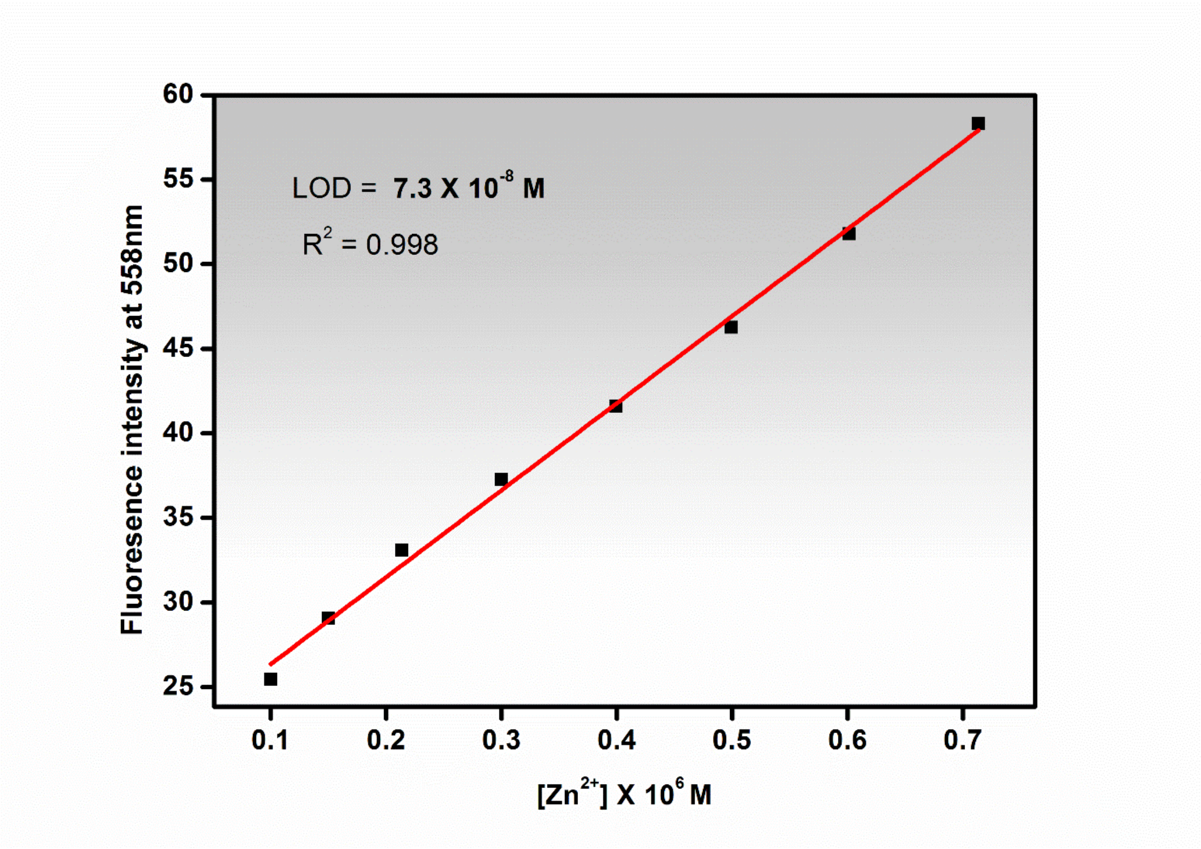

Figure S16. Determination of the detection limit of **IIED** in presence of Zn^{2+} for emission at 558 nm.

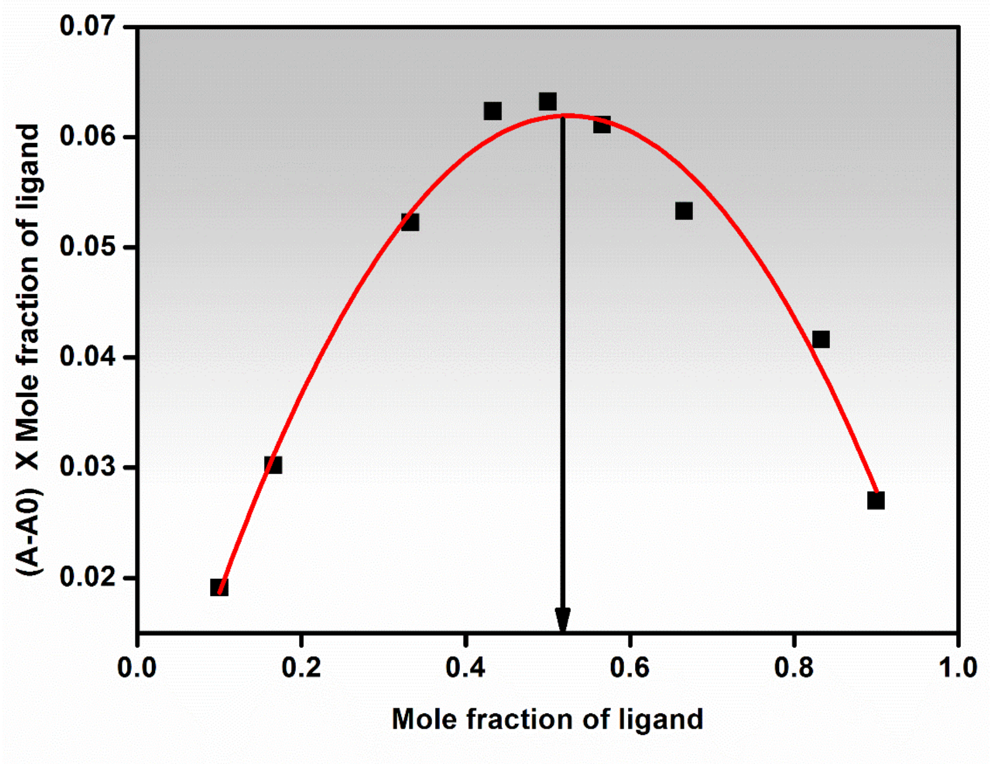

Figure S17. Job's plot for the identification of stoichiometry using absorbance values.

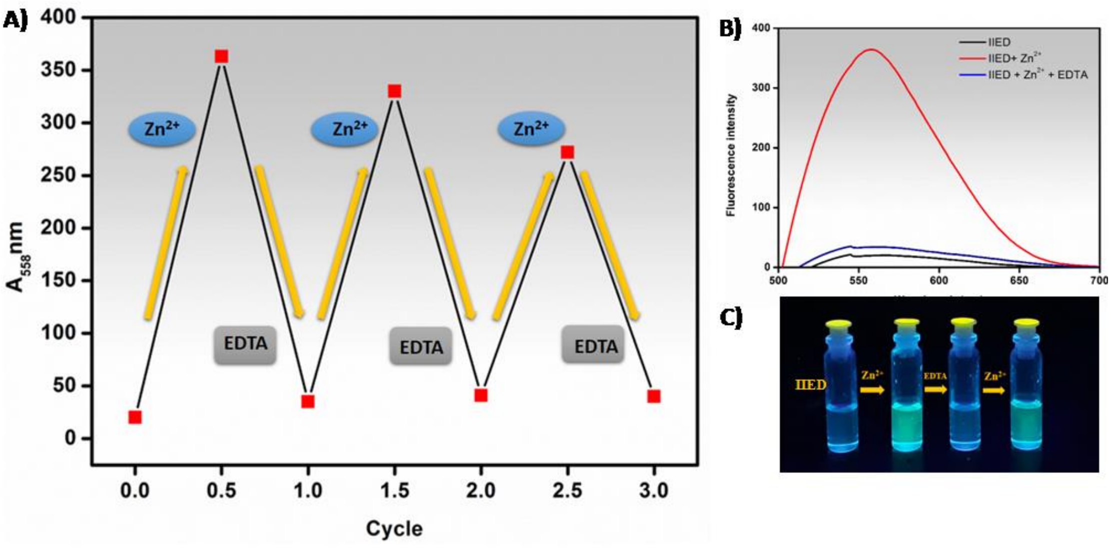

Figure S18. (A) The reversible colorimetric switch by alternate addition of Zn^{2+} and EDTA in (3:7 v/v) HEPES buffer : DMSO medium. (B) Emission spectrum of **IIED** shows reversibility towards Zn^{2+} in presence of EDTA in (3:7 v/v) HEPES buffer : DMSO medium. (C) Visual change of **IIED** under UV light in the presence of Zn^{2+} and EDTA.

Table S1. Time resolved fluorescence data

**Parameters**	**IIED**	**IIED + Zn^{2+}**
χ^2	**1.12**	**1.018**
α_1	**0.02**	**0.0321**
α_2	**0.007**	**0.0321**
α_3	**0.07**	**0.0229**
τ_1	**1.42 ns**	**1.35 ns**
τ_2	**5.09 ns**	**5.57 ns**
τ_3	**0.26 ns**	**0.32 ns**
τ_{avg}	**2.74 ns**	**3.65 ns**

k_r	**0.072 × 10^9** sec^{-1}	**0.189 × 10^9**sec^{-1}
k_{nr}	**0.29 × 10^9**sec^{-1}	**0.0849 × 10^9** sec^{-1}
